# Nutritional therapy for the prevention of post-intensive care syndrome

**DOI:** 10.1186/s40560-024-00734-2

**Published:** 2024-07-29

**Authors:** Taku Oshima, Junji Hatakeyama

**Affiliations:** 1 Institute for Advanced Academic Research, Chiba University, 1-33 Yayoi-Cho, Inage-Ku, Chiba-Shi, Chiba 263-8522 Japan; 2 Department of Emergency and Critical Care Medicine, Chiba University Graduate School of Medicine, 1-8-1 Inohana, Chuo-Ku, Chiba City, Chiba 260-8677 Japan; 3 Department of Emergency and Critical Care Medicine, Osaka Medical and Pharmaceutical University, 2-7, Daigaku-machi, Takatsuki, Osaka 569-8686 Japan

**Keywords:** ICU-acquired weakness, PICS, Malnutrition, Indirect calorimetry, Protein nutrition

## Abstract

Post-intensive care syndrome (PICS) is a triad of physical, cognitive, and mental impairments that occur during or following the intensive care unit (ICU) stay, affecting the long-term prognosis of the patient and also the mental health of the patient’s family. While the severity and duration of the systemic inflammation are associated with the occurrence of ICU-acquired weakness (ICU-AW), malnutrition and immobility during the treatment can exacerbate the symptoms. The goal of nutrition therapy in critically ill patients is to provide an adequate amount of energy and protein while addressing specific nutrient deficiencies to survive the inflammatory response and promote recovery from organ dysfunctions. Feeding strategy to prevent ICU-AW and PICS as nutrition therapy involves administering sufficient amounts of amino acids or proteins later in the acute phase after the hyperacute phase has passed, with specific attention to avoid energy overfeeding. Physiotherapy can also help mitigate muscle loss and subsequent physical impairment. However, many questions remain to be answered regarding the potential role and methods of nutrition therapy in association with ICU-AW and PICS, and further research is warranted.

## Introduction

Critically ill patients undergo severe stress due to their critical illness, generally enhancing their metabolic demands to support the inflammatory response [1]. Such metabolic demand is sustained by a catabolic response to use endogenous energy supply, especially during the acute phase of the illness [1, 2]. Systemic inflammation, catabolic response, and prolonged immobilization during the treatment cause polyneuropathy and muscle degeneration, leading to the progressive deterioration of physical function known as intensive care unit-acquired weakness (ICU-AW) [3, 4]. Patients with ICU-AW are at risk of developing post-intensive care syndrome (PICS) when combined with cognitive or psychological impairments [5], and malnutrition is considered one of the risk factors for the development and progression of the syndromes [5]. In this review, we will focus on the impact of nutrition therapy on preventing ICU-AW and the subsequent PICS development, as well as the practical issues to implement current suggestions in clinical practice.

### Post-intensive care syndrome

**Fig. 1:**
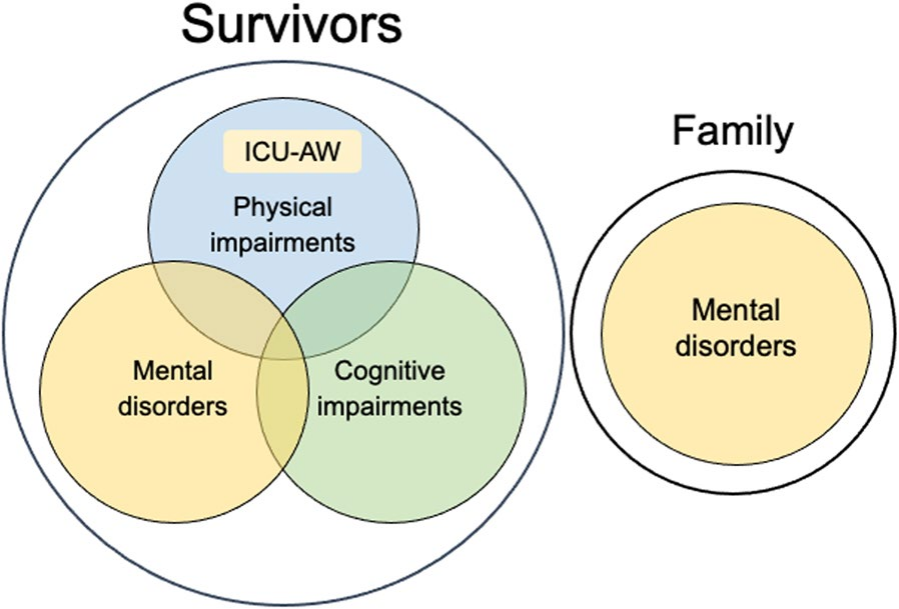
Conceptual components of post-intensive care syndrome (PICS) and its effect on the patients and their families: survivors of critical illness frequently experience intensive care unit-acquired weakness (ICU-AW) or global physical impairment as a result of muscle and peripheral nerve degeneration and develop PICS when complicated with cognitive dysfunction and mental disorder. Patients’ families also may develop mental disorders as a result of severe mental stress during hospitalization

Post-intensive care syndrome (PICS) is a collective term that describes a triad of physical, cognitive, and mental impairments that occur during the intensive care unit (ICU) stay or following ICU or hospital discharge, affecting not only the long-term prognosis of a patient recovering from severe injury, but also the mental health of the patient’s family (Fig. 1) [6, 7]. More recently, ICU-acquired chronic pain has also been considered one of the hallmarks of PICS, as it exacerbates physical, mental, and cognitive impairments and vice versa [8]. In an epidemiological study in Japan, the prevalence of PICS six months after ICU discharge was 64%, with 32% having physical impairment, 38% having cognitive impairment, 15% having mental impairment, and 18% having multiple functional impairments, indicating the complexity of PICS symptoms [9]. The prevalence of PICS in patients with coronavirus disease 2019 (COVID-19) requiring mechanical ventilation at six months after ICU discharge in Japan was 58.6%, with cognitive impairment being the most common (46.6%), followed by mental impairment (31.9%) and physical impairment (21.9%), indicating that quality of life (QOL) deteriorated as functional impairment developed [10]. The deterioration of patients’ QOL due to critical illness has a profound impact on their employment status. Survivors of severe disease conditions with physical and mental impairment experience economic stress from worsening employment conditions, such as unemployment, occupational changes, and reduced working hours [11, 12]. Thus, PICS is an international problem in the field of intensive care, and there is an urgent need to implement PICS prevention measures.

Risk factors for developing PICS consist of patient, disease, treatment, and environmental factors. There are many reported risk factors for ICU-AW, or factors leading to physical impairment, which can be classified into avoidable and non-avoidable risk factors. Non-avoidable factors include the severity of the critical illness and systemic inflammatory response syndrome, sepsis, multi-organ failure, hyperlactatemia, duration of mechanical ventilation, being a female and having old age [13, 14]; avoidable factors include hyperglycemia associated with systemic inflammation, beta-receptor stimulants, corticosteroids, neuromuscular blocking agents, antimicrobials such as aminoglycosides and vancomycin, and prolonged sedative use [15–18]. Prolonged use of sedatives is one of the most significant risk factors for delirium and cognitive impairment in the ICU and, at the same time, the biggest risk factor for cognitive impairment after ICU discharge [19]. Cognitive impairment already present prior to ICU admission is also an important risk factor [20] while other risk factors include environmental factors such as closed rooms, lighting at night, noise, and background noise [21, 22]. Risk factors for mental disorders include a history of psychiatric illness prior to ICU admission, alcoholism and prolonged opioid use, and unpleasant or delusional memories and memory lapses in the ICU [23, 24]. Deep sedation tends to produce memory impairment and unpleasant memories [25–27], and benzodiazepines have been implicated in mental disorders because of their amnesic effects [28].

### Role of nutrition in the prevention of post-intensive care syndrome

PICS consists of the symptoms of physical function impairment known as ICU-AW [3], combined with cognitive and psychological impairment during and after the treatment in the ICU [5]. While the severity and duration of the systemic inflammation associated with the critical illness have a significant impact on the onset of ICU-AW, malnutrition and immobility during the treatment can exacerbate the symptoms [3, 5]. ICU-AW is known to occur as a result of the various myopathy and neuropathy responses induced by the critical illness [3] and is also associated with muscle loss occurring as a result of the catabolic response to utilize protein as an energy source and also as components of the inflammatory system to sustain the humoral response [29]. Muscle loss is observed early in the course of the critical illness, reflecting the association with metabolic alterations, especially during the first week when up to 20% of the muscle mass can be lost in accordance with the severity of the disease characterized by multiple organ dysfunction [30, 31]. ICU-AW is associated with prolonged physical impairment [32], and the severity of the symptoms has a direct impact on late-phase mortality [33]. Adequate energy and protein nutrition are supposed to prevent excessive muscle mass loss and physical function impairment during critical illness, while malnutrition contributes to muscle wasting and impaired physical function. Although the catabolic response is not sufficiently attenuated by supplying exogenous energy sources as nutrition [29], it is suggested that it helps maintain the small amount of anabolic response, mitigating excessive muscle mass loss [34]. Early mobilization can also help stimulate muscle synthesis when combined with sufficient nutrition provision [35].

### Nutrition for critically ill patients: general recommendations

The goal of nutrition therapy in critically ill patients is to provide an adequate amount of energy and protein while addressing specific nutrient deficiencies to sustain the inflammatory response and promote recovery from organ dysfunctions [36]. Energy provision targets should be determined according to energy expenditure measured by indirect calorimetry by carefully accounting for the metabolic alterations during the early phase of critically ill patients [37]. Since the endogenous energy production as a part of the catabolic response will not be sufficiently suppressed by administering nutrition [1], energy provision should be initiated from a small amount and carefully progressed to the targets to avoid overfeeding [36]. Protein supplementation should also be according to defined targets [29], although the optimal dosing remains somewhat controversial. While high-protein supplementation of 1.0 ~ 2.0 g/kg body weight is expected to reduce muscle mass loss and preserve physical function [38, 39], a negative impact is reported in the mortality of the septic population. According to a secondary analysis of recent RCTs, early protein supplementation induces an abundance of amino acids within the blood, suppressing autophagy that helps remodel injured tissues, including the skeletal muscle [40]. Thus, a reduced amount of protein is recommended (< 1.0 g/kg body weight) for this specific population [41]. Early enteral nutrition is recommended to preserve gut integrity and reduce the risk of infectious complications [42, 43], while parenteral nutrition should also be considered for those with malnutrition [39, 44, 45].

### Clinical trials of nutritional therapy as a PICS measure

No major clinical trials have directly investigated the effect of acute phase nutritional interventions with PICS as the primary outcome, and evidence for mitigating PICS by nutrition therapy has not been established to date (Table 1). One-year follow-up of the EDEN trial demonstrated no difference in physical or cognitive function between the full-feeding and trophic-feeding groups [46]. In the subanalysis of the EPaNIC trial, the incidence of ICU-AW was higher in the early PN group, which may be attributed to the suppression of autophagy due to the abundance of circulating amino acids, resulting in delayed remodeling of damaged tissues, thus impaired organ and physical dysfunctions [15]. On the other hand, the early PN group in the Early PN Trial had less muscle wasting and fat loss as the secondary endpoints. The difference in the effect may be related to the fact that the patients in the Early PN Trial were not overfed, while the comorbidities in the early PN group in the EPaNIC trial were considered as the result of severe overfeeding [47].

**Table 1:** RCT combining acute nutrition therapy and rehabilitation with physical function as the outcome

Author, year	Eligible patients	Nutrition and rehabilitation collaboration group			Control group		Primary outcomes and results	ICU-AW incidence/MRS score and results	Other physical functions and results
		n	Nutritional therapy	Rehabilitation	n	Treatment			
Nakano, 2021	Admitted to ICU	56	**Amount of energy**						
			MUST ≥ 4: 30 kcal/kg/day MUST < 4: 20 kcal/kg/day	Standard rehabilitation plus NMSE according to IMS	45	Standard nutritional therapy and rehabilitation	Significantly lower rate of decrease in thigh muscle mass by CT on day 10 of ICU admission	No difference in MRC score at ICU discharge	No difference in grip strength, FSS-ICU or BI at ICU discharge
			**Protein Content** 1.8 g/kg/day as EN, with supplementational PN						
de Azevedo, 2021	MV for ≥ 72 h	87	**Days 3 and 4** Energy dose of 50–70% of REE by indirect calorimetry and protein dose of 0.8–1.0 g/kg/day			**Days 3 and 4** Energy dose of 50–70% of REE by indirect calorimetry and protein dose of 0.8–1.0 g/kg/day			
			**Days 5 and 6** Energy dose of 80% of REE by indirect calorimetry and protein dose of 2.0–2.2 g/kg/day	Standard rehabilitation plus cycle ergometer for 15 min twice a day	94	**Days 5 and 6** Energy dose of 80% of REE by indirect calorimetry and protein dose of 1.4–1.5 g/kg/day	SF-36 PCS significantly increased in intervention group at 3 and 6 mos	No difference in incidence of ICU-AW using a grip strength meter at ICU discharge or after 21 days of ICU stay	NA
			**Days 7 to 10** If protein dose does not reach the target dose, add to PN			**Days 7 to 10** If protein dose does not reach the target dose, add to PN			
Zhou, 2022	Expected ICU stay ≥ 72 h At least three of the following orders: “open and/or close your eyes”, “look at me”, “put out your tongue”, “nod your head”, and “raise your eyebrows”; BI ≥ 70 at 2 weeks before ICU admission	50	EN started within 48 h of ICU admission	Mobilization started within 24 h of ICU admission, twice daily, 20–30 min/session, until ICU discharge	50	Standard ICU care or physician experience treatment	Significantly lower incidence of ICU-AW at ICU discharge in the intervention group	No difference in MRS score at ICU discharge	Significantly better of BI in the intervention groups upon ICU discharge
Verceles, 2023	Age ≥ 50 years with requiring MV BI ≥ 70 before ICU admission	16	Protein dose of 1.75 g/kg/day	Standard rehabilitation plus NMSE (30 min twice daily for 14 days or until ICU discharge; stimulation duration 300 ms on/off for 10 s)	23	Standard ICU Care	Significantly lower rate of decrease in thigh muscle mass by CT on ICU day 14 in the intervention group	NA	NA

*BI* Barthel index, *CT* computed tomography, *EN* enteral nutrition, *FSS-ICU* Functional Status Score for the ICU, *ICU* intensive care unit, *ICU-AW* ICU-acquired weakness, *IMS* Intensive Care Unit Mobility Scale, *MRC* Medical Research Council, *MUST* malnutrition universal screening tool, *MV* mechanical ventilation, *NA* not available, *NMSE* neuromuscular electrical stimulation, *PN* parenteral nutrition, *RCT* randomized controlled trial, *REE* resting energy expenditure, *SF-36 PCS* short form-36 physical component summary

The importance of providing nutrition therapy in coordination and collaboration with early mobilization and rehabilitation has been discussed recently to have benefits in the long-term prognosis of critically ill patients [5]. Studies have reported advantages of adding neuromuscular electrical stimulation (NMSE) to the usual rehabilitation program, the adjustment of energy and protein administration according to the evaluation of malnutrition using the malnutrition universal screening tool (MUST), physiotherapy adjustment according to activity level in the ICU while providing early enteral nutrition, and exercise ergometry while loading protein as nutrition therapy [48–51]. Although combining rehabilitation and nutritional therapy has shown some improvement in physical function and maintenance of muscle mass and strength in these studies, the overall effect is difficult to evaluate because of the various outcomes and evaluation tools involved. The intensity of rehabilitation interventions and nutritional therapy during ICU admission should be modified according to the patient’s physical condition and nutritional status before admission to the ICU. A recent retrospective study by Liu et al. demonstrated that early mobilization within 3 days, or more specifically the first 2–4 days of ICU admission, was associated with better clinical outcomes in septic patients [52]. The Japanese Society for Critical Care Medicine recently published the first evidence-based guideline on rehabilitation for critically ill patients. The guideline emphasizes the importance of implementing standardized rehabilitation protocol to provide daily rehabilitation programs, including dysphagia management and promoting multidisciplinary collaboration during and following ICU treatment [53]. There is no doubt that nutritional therapy and rehabilitation are both important; however, supportive evidence is lacking, and future clinical studies should have sufficient focus on this issue.

**Fig. 2:**
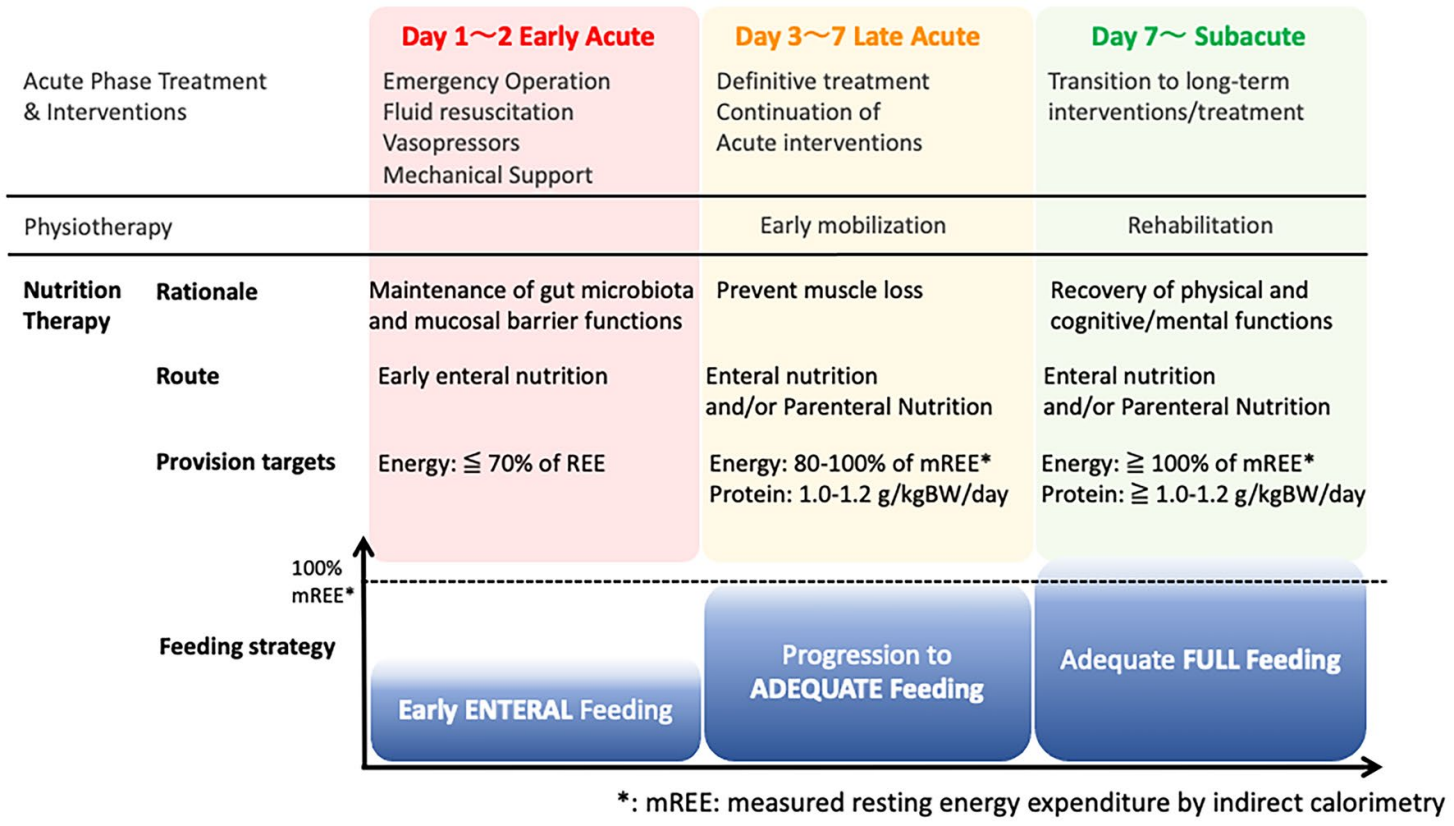
Early feeding strategy to prevent PICS in critically ill patients: early intervention with nutrition and physiotherapy has been suggested as a measure to prevent PICS in critically ill patients. Enteral nutrition should be initiated early to test for feeding tolerance and progress to meet the energy needs measured by indirect calorimetry after initial stabilization . Protein targets should also be determined for individual patients. Physiotherapy can start as early passive mobilization or electrical muscle stimulation (EMS) and gradually progress to individualized rehabilitation. Such intervention should be planned according to the course of the critical illness and the phase of treatment

Based on the current evidence and guideline recommendations, a tentative strategy to avoid ICU-AW and PICS should involve the early initiation of both nutrition therapy and mobilization, gradually progressing to full-energy feeding and comprehensive rehabilitation programs during the later periods in the acute phase (Fig. 2). Enteral nutrition, if not contraindicated, should be tested for tolerance by starting at a trophic amount as soon as the systemic circulation is stabilized, usually when fluid and catecholamine doses are titrated. Gradual progression to energy and protein goals via enteral or intravenous routes, as well as early mobilization, may begin in the later acute phase when the initial inflammatory response has resolved by acute phase treatments, with specific attention to avoid energy overfeeding. Nutrition therapy and rehabilitation can be further advanced when the patients have survived the critical illness to be weaned off mechanical and medical organ support therapies.

### Practical issues regarding PICS preventive nutrition therapy

The difficulty in the prevention of PICS and the underlying pathology of ICU-AW lies in the fact that both are difficult to recognize until the symptoms have developed and patients are stabilized enough to weaned off sedatives and mechanical organ support therapies [5]. By then, the symptoms have progressed to the degree that prolonged ICU and hospital stays are inevitable. It is also difficult to predict which patients would become candidates for PICS as much as it is difficult to foresee the recovery or the deterioration of critically ill patients during the first days of treatment. This is why early intervention is recommended in all critically ill patients. Energy targets should be based on the EE measurements by indirect calorimetry; however, the optimal timing to start and progress to full-energy feeding is yet to be elucidated [37]. Protein targets are also controversial, as previously described.

Feeding the critically ill patients efficiently and safely is another issue. While early enteral nutrition is universally recommended in this population unless the enteral route is contraindicated, enteral feeding intolerance frequently prevents sufficient nutrition intake, although the consensus for the definition of feeding intolerance has not been established [54]. It can also lead to harm in hemodynamically unstable patients in the form of mesenteric ischemia [55, 56]. Monitoring becomes crucial in successful feeding procedures, to avoid overfeeding and underfeeding, and to account for symptoms of enteral feeding intolerance. Individualized feeding protocols implemented by dieticians and nurses as multidisciplinary teams have shown promising results in increasing the adequacy of the feeding regimen, enhancing muscle protein synthesis, and promoting rehabilitation.

## Conclusion

Post-intensive care syndrome is a growing threat to critically ill patients, and prevention is of utmost importance, given the difficulty of treating them after diagnosis. There are still many questions to be answered regarding the potential role of nutrition therapy. The role of nutrition therapy and the difficulty of implementing preventive strategies in clinical practice should be recognized, and further studies to optimize the interventions are warranted.

## Data Availability

Not applicable.
